# Configurable Multi-Layer Perceptron-Based Soft Sensors on Embedded Field Programmable Gate Arrays: Targeting Diverse Deployment Goals in Fluid Flow Estimation †

**DOI:** 10.3390/s25010083

**Published:** 2024-12-26

**Authors:** Tianheng Ling, Chao Qian, Theodor Mario Klann, Julian Hoever, Lukas Einhaus, Gregor Schiele

**Affiliations:** Intelligent Embedded Systems of Computer Science, University of Duisburg-Essen, 47057 Duisburg, Germany; chao.qian@uni-due.de (C.Q.); gregor.schiele@uni-due.de (G.S.)

**Keywords:** Internet of Things, embedded systems, fluid flow estimation, soft sensors, quantized neural networks, quantization-aware training, hardware–software co-design, embedded FPGA-based acceleration, energy efficiency

## Abstract

This study presents a comprehensive workflow for developing and deploying Multi-Layer Perceptron (MLP)-based soft sensors on embedded FPGAs, addressing diverse deployment objectives. The proposed workflow extends our prior research by introducing greater model adaptability. It supports various configurations—spanning layer counts, neuron counts, and quantization bitwidths—to accommodate the constraints and capabilities of different FPGA platforms. The workflow incorporates a custom-developed, open-source toolchain *ElasticAI.Creator* that facilitates quantization-aware training, integer-only inference, automated accelerator generation using VHDL templates, and synthesis alongside performance estimation. A case study on fluid flow estimation was conducted on two FPGA platforms: the AMD Spartan-7 XC7S15 and the Lattice iCE40UP5K. For precision-focused and latency-sensitive deployments, a six-layer, 60-neuron MLP accelerator quantized to 8 bits on the XC7S15 achieved an MSE of 56.56, an MAPE of 1.61%, and an inference latency of 23.87 μs. Moreover, for low-power and energy-constrained deployments, a five-layer, 30-neuron MLP accelerator quantized to 8 bits on the iCE40UP5K achieved an inference latency of 83.37 μs, a power consumption of 2.06 mW, and an energy consumption of just 0.172 μJ per inference. These results confirm the workflow’s ability to identify optimal FPGA accelerators tailored to specific deployment requirements, achieving a balanced trade-off between precision, inference latency, and energy efficiency.

## 1. Introduction and Related Work

Soft sensors, functioning as virtual instruments, utilize algorithms and computational models to estimate unobservable or impractical values by processing data derived from physical sensors [1,2]. In the *Internet of Things* (IoT) domain, where obtaining direct measurements can often be impractical or economically burdensome, soft sensors have been increasingly adopted as a cost-effective alternative.

Early implementations of soft sensors mainly utilized physical models [3] or statistical estimators [4,5]. While these methods are straightforward, their ability to handle the complex, nonlinear relationships inherent in real-world scenarios is often limited. The emergence of *Deep Learning* (DL) has significantly advanced soft sensor development, enabling the modeling of intricate dependencies and patterns in sensor data [6]. *Neural Networks* (NNs) architectures, including *Multi-Layer Perceptron* (MLP), *Convolutional Neural Networks* [7], *Recurrent Neural Networks* [8], and *Graph Neural Networks* [9], have extended the scope of soft sensors, with applications spanning industrial automation, environmental monitoring, and beyond [10,11], particularly when deployed on scalable Cloud-based platforms [6,10].

While Cloud-based deployments offer scalable resources and easy accessibility, they are not without drawbacks, particularly for real-time IoT applications. Systems that rely on Cloud processing are susceptible to network instability, which can lead to delays, thus compromising the timeliness of data-driven responses in critical applications [12]. Additionally, the limited data transmission rates of many wireless sensor networks, such as LoRaWAN with a maximum rate of 50 kbps, restrict real-time, high-resolution raw data transmission, particularly in infrastructure-limited environments [12]. These constraints underscore the value of deploying soft sensors directly on IoT devices, where computations are performed locally, reducing latency and enhancing data privacy by minimizing network dependency.

Recent advancements have shifted the focus toward deploying DL models on embedded hardware to address these constraints. Aguasvivas Manzano et al. [13] and Flores et al. [14] demonstrated the feasibility of implementing soft sensors on ARM Cortex-M4 and ESP32 *Microcontroller Unit* (MCU), achieving low latency and power consumption. However, the limited computational capacity of MCUs restricts their ability to support complex models. In addition, Tiny IoT devices, typically constrained by size, power, and cost, may not be able to be equipped with embedded GPUs [15]. Balaji et al. [16] explored *AMD XC7A15T Field Programmable Gate Array* (FPGA)-based accelerators for wearable devices, highlighting FPGAs’ capability to offer high performance with tailored resource utilization.

Our previous work [17] has explored the deployment of DL-based soft sensors directly on IoT devices, focusing on MLP-based soft sensors for fluid flow estimation. By performing analysis on-device, we mitigate network dependencies but face new challenges due to the limited computational resources of IoT devices. These constraints necessitate model optimization, a process we addressed through quantization techniques. Specifically, we implemented quantized MLP models on resource-constrained hardware, namely a low-power *ARM Cortex-M0+* MCU and an embedded *AMD Spartan-7 XC7S15* FPGA. Using our custom *ElasticAI.Creator* toolchain (https://github.com/es-ude/elastic-ai.creator accessed on 13 November 2023), we achieved up to a 28.44× increase in inference speed for FPGA-deployed models over MCU-based implementations using *TensorFlow Lite Micro* (https://github.com/tensorflow/tflite-micro accessed on 13 November 2023), meeting real-time requirements. However, model precision suffered due to the limitations of fixed-point quantization for the FPGA implementation, motivating further improvements. In a follow-up study [18], we improved model precision through integer-only quantization, which aligns quantization parameters with data distributions across tensors. This refinement increased precision by up to 9.7% while pipelined matrix multiplications reduced inference time by 9.39%, with minimal energy cost increase.

However, despite these advances, the ability to customize model configurations and adapt to varying deployment goals remained limited. Depending on the application, embedded DL-based soft sensors are often subject to diverse deployment goals—such as achieving high precision, minimal inference latency, low power consumption, and optimal energy efficiency. Meeting these goals involves balancing factors like model complexity (layer count, neuron count), quantization bitwidth, and hardware platform selection, all of which directly impact resource usage and computational demand. For instance, increasing model complexity may improve precision but can also lengthen inference time and increase power draw, which could be suboptimal for low-power deployments. Similarly, reducing quantization bitwidth lowers resource consumption but may compromise model precision.

To address these challenges systematically, this study investigates the following research question: Which configuration factors—including layer count, neuron count, quantization bitwidth, and FPGA type—most significantly impact the deployment of MLP-based soft sensors on embedded FPGAs for goals such as precision, inference time, power consumption, and energy efficiency? By exploring this question, we provide a workflow for evaluating the trade-offs required to meet specific deployment objectives in resource-limited environments. We extend our previous work [18] with the following contributions:

- **Increased Model Configurability and Complexity**: We enhance the flexibility of MLP accelerators for embedded FPGAs by enabling customizable configurations of layer count, neuron count, and quantization bitwidth. This configurability allows developers to adapt models to different deployment requirements, balancing metrics like precision, inference speed, and resource usage.
- **Cross-Platform FPGA Support and Optimized Toolchain Integration**: We introduce an open-source, user-friendly toolchain that integrates *Quantization-Aware Training* (QAT), integer-only inference, automated accelerator generation through VHDL templates, along with synthesis and performance estimation across diverse FPGA platforms. This toolchain simplifies deployment, making it accessible for users without deep FPGA expertise to optimize and deploy models across multiple hardware configurations.
- **Case Study in Fluid Flow Estimation**: Using fluid flow estimation as a case study, we validate our configurable MLP-based soft sensors on two FPGA platforms: the AMD Spartan-7 XC7S15 and the Lattice iCE40UP5K. Our experiments highlight the trade-offs across different configurations, providing insights into the effects of varying model complexity on precision, inference time, power, and energy consumption.

The remainder of this paper is organized as follows: Section 2 presents the system architecture and requirements for on-device soft sensors. Section 3 introduces foundational concepts, including MLP models and integer-only quantization. Section 4 describes our software–hardware co-design approach for efficient FPGA deployment, leveraging QAT and integer-only inference. Section 5 introduces our workflow integrated with an open source toolchain, streamlining model deployment and evaluation across FPGA platforms. Section 6 provides an overview of the testbed platforms used in our study, comparing the XC7S15 and iCE40UP5K FPGAs. Section 7 details the experimental setup, including case study, datasets, and evaluation metrics. Section 8 presents our findings and offers insights into trade-offs across configurations. Finally,  Section 9 summarizes key insights and suggests directions for future research.

## 2. System Architecture

**Figure 1:**
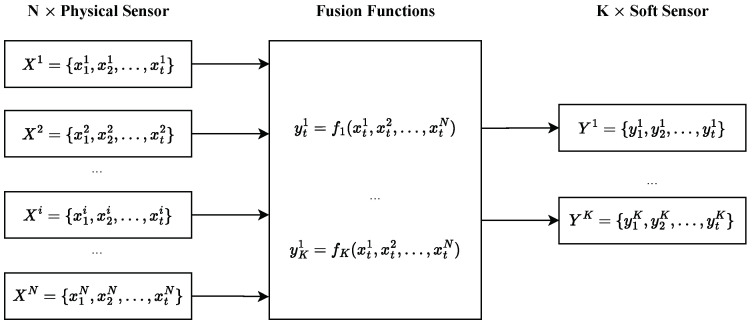
General system architecture of soft sensors.

Our soft sensor system employs a modular architecture to accommodate diverse deployment goals. As illustrated in Figure 1, the system integrates data from *N* physical sensors to produce *K* independent soft sensor outputs. Each sensor $X^{i}\;=\;\left\{x_{1}^{i},x_{2}^{i},\ldots ,x_{t}^{i}\right\}$, where i∈{1,…,N}, captures a discrete time series sampled at fixed intervals *T*.

The architecture employs *K* fusion functions, $f_{1},f_{2},\ldots ,f_{K}$, to transform multi-sensor inputs into distinct outputs $Y^{k}\;=\;\left\{y_{1}^{k},y_{2}^{k},\ldots ,y_{t}^{k}\right\}$, effectively converting *N* -dimensional inputs into *K* -dimensional outputs. This design reduces data volume when K<N, minimizing transmission loads for remote monitoring applications. To meet real-time requirements, each fusion operation must be completed within the sampling period *T*.

In our implementation, these fusion functions are realized using MLP models initially trained in the Cloud. After achieving satisfactory performance, the models are deployed on IoT devices as on-device soft sensors. This approach eliminates the need for continuous Cloud connectivity, reducing latency and energy consumption while enabling real-time processing.

## 3. Fundamentals

This chapter introduces the foundational principles of MLP architecture and integer-only quantization, which form the basis for the efficient deployment of MLP-based soft sensors on embedded FPGAs.

### 3.1. Multi-Layer Perceptron Architecture

**Figure 2:**
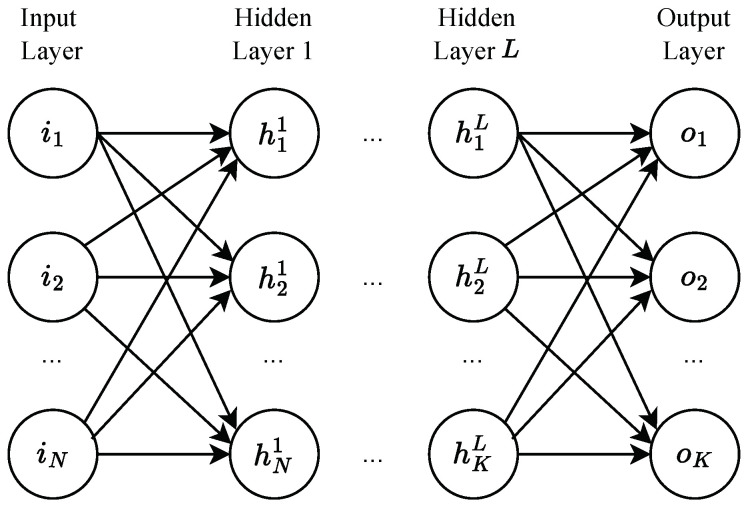
Architecture of an MLP model.

The MLP is composed of multiple layers of interconnected artificial neurons [19]. It is widely used for modeling complex, nonlinear relationships in data. As illustrated in Figure 2, an MLP consists of three main types of layers: (1) an input layer that maps input features to the first hidden layer, (2) hidden layers that process intermediate representations to capture nonlinear patterns, and (3) an output layer that generates the final predictions.

Connections between neurons in adjacent layers are mathematically represented using matrix operations. Specifically, the inputs *X* to each layer are multiplied by a weight matrix *W*, added to a bias vector *B*, and passed through an activation function σ, as shown in Equation (1). In this work, we employ the *Rectified Linear Unit* (ReLU) activation function (Equation (2)) [20] for its computational efficiency in FPGA deployment.

(1) $$A=\sigma \left(Y\right)=\sigma \left(XW^{T}+B\right)$$

(2) $$\mathrm{ReLU}\left(y\right)=\left\{\begin{array}{cc} y & \mathrm{if}\;y>0\\ 0 & \mathrm{otherwise} \end{array}\right.$$

To meet varied deployment goals, this study allows for adjustments in model complexity (i.e., layer count and neuron count), providing flexibility for balancing precision, inference speed, and resource usage on FPGAs. Here, *layer count* refers to the total number of layers in the MLP model (e.g., a four-layer MLP includes one input layer, one output layer, and two hidden layers), while *neuron count* denotes the number of neurons per hidden layer. For simplicity, all hidden layers are configured with the same neuron count by default, but this setup can be customized based on specific application requirements.

### 3.2. Integer-Only Quantization

NNs, including MLPs, are typically trained with 32-bit floating-point (FP32) values in R to represent model parameters and activations [21]. However, deploying models on resource-constrained hardware requires reducing numerical precision to minimize memory usage and accelerate inference [22]. Integer-only quantization is one of the key techniques in achieving this [23,24], converting values from the continuous domain R into a discrete, quantized domain Q, which significantly optimizes both memory footprint and processing speed for embedded systems.

Consider a tensor *X*, composed of real-valued elements *x*, and its quantized representation $X_{q}$. As depicted in Equation (3), the scale factor *S*, which is a floating-point parameter, defines the relationship between *X* and $X_{q}$. In addition, the zero point *Z* is an integer parameter representing zero in *X*. The rounding operation approximates *x* to the nearest integer, and the clamp function ensures $x_{q}$ remains within the bounds of a *b*-bit signed integer $\left[-2^{b-1},2^{b-1}-1\right]$. De-quantization, detailed in Equation (4), reverses this process, converting $X_{q}$ back into an approximate real-valued tensor $X^{\prime }$, using the same scale factor *S* and zero point *Z*.

(3) $$\begin{array}{cc} X\mapsto X_{q} & =\mathrm{clamp}\left(\mathrm{round}\left(\frac{X}{S}\right)+Z,-2^{b-1},2^{b-1}-1\right) \end{array}$$

(4) $$\begin{array}{cc} X_{q}\mapsto X^{\prime } & =S\cdot (X_{q}-Z) \end{array}$$

We utilize integer-only quantization with adaptive parameters, as per [23]. This method dynamically computes the scaling factor *S* and zero point *Z*, customizing them to each tensor’s distribution. Equations (5) and (6) illustrate how these parameters are computed based on the tensor’s observed minimum (α) and maximum (β) values. This adaptive approach ensures a more efficient numerical representation, especially for data distributions that are not symmetric around zero. This enhances model performance by minimizing quantization errors between *X* and $X^{\prime }$ and optimizes bit precision utilization.

(5) $$\begin{array}{cc} S & =\frac{\alpha -\beta }{2^{b}-1} \end{array}$$

(6) $$\begin{array}{cc} \;Z & =\mathrm{clamp}\left(\mathrm{round}\left(\left(2^{b-1}-1\right)-\frac{\alpha }{S}\right),-2^{b-1},2^{b-1}-1\right) \end{array}$$

## 4. Software–Hardware Co-Design

To efficiently deploy configurable MLP-based soft sensors on embedded FPGAs, this chapter details our integrated software and hardware co-design approach. Building on the principles of MLP architecture and integer-only quantization discussed in Section 3, we implement a customized QAT pipeline within the PyTorch framework and develop integer-only inference optimizations for embedded FPGA deployment. This co-design ensures that MLP architectures can be configured with varying complexity levels and quantization parameters, enabling them to meet diverse deployment goals.

### 4.1. Customized Software Implementation

#### 4.1.1. Quantization-Aware Training

In our work, we implement a custom QAT pipeline in PyTorch, tailored specifically to enhance integer-only quantization precision in MLP-based soft sensors. QAT minimizes precision loss by modeling quantization effects during training, allowing NNs to adjust for reduced bitwidth representations [23]. To accommodate non-differentiable rounding in back-propagation, we incorporate the *Straight-Through Estimator* to approximate gradients for non-differentiable operations [25].

As detailed in Table 1, our example implementation uses a four-layer MLP model to demonstrate the key quantization objects and parameters. These include the inputs *X*, weights $W^{1}$, biases $B^{1}$, outputs $Y^{1}$, and activations *A* for the hidden layer 1, and weights $W_{2}$, biases $B_{2}$ for the hidden layer 2, and final outputs $Y^{2}$ for the output layer. The quantization parameters of each quantization object are dynamically updated after each training batch, aligning them with the actual data distribution to minimize quantization errors. The specific quantization parameters for each object are summarized in Table 1. For example, the quantization scale factor for the hidden layer 1’s inputs is $S_{X}$, with a corresponding zero point of $Z_{X}$. Notably, all biases have no zero points for computational simplicity. Notably, although our implementation supports mixed-precision quantization, all quantization objects in this study are uniformly quantized at the same bitwidth for model consistency and simplified analysis.

In our study, we adapt the ReLU function’s implementation. During QAT, we employ PyTorch’s standard torch.nn.functional.relu function (https://pytorch.org/docs/stable/generated/torch.nn.functional.relu.html accessed on 24 December 2024). For subsequent integer-only inference, the quantization parameters of the ReLU function’s inputs are inherited from the outputs of the fully connected layer in the same hidden layer, i.e., scale factor $S_{Y^{1}}$ and zero point $Z_{Y^{1}}$. In addition, we use the same quantization parameters for the outputs of the ReLU function to ensure uniformity and coherence in quantization across inputs and outputs. Similarly, for the inputs of the second hidden layer, the inputs $Y^{1}$ inherit their quantization parameters directly from the first hidden layer’s outputs $Y^{1}$. This approach maintains a consistent and coherent quantization method throughout the model, facilitating the integrity of the quantization process across varying layers.

#### 4.1.2. Enhanced Integer-Only Inference

Although some embedded FPGAs support floating-point operations, these typically incur higher resource usage and power consumption compared to integer arithmetic [26]. Our study employs integer-only inference, where all computations and inter-layer data transfers are conducted using integer arithmetic, ensuring efficient deployment on resource-constrained embedded FPGAs.

##### Integer-Only Fully Connected Layer

We employ the integer-only fully connected layer calculation method proposed by Benoit et al. [24], starting from the first hidden layer of our model. This computation leverages quantization parameters determined during the QAT phase, as shown in Equation (7). To streamline the calculation, we approximate the bias term $S_{B^{1}}B_{q}^{1}$ as $S_{X}S_{W^{1}}B_{q}^{1*}$, where the originally 8-bit quantized bias is adjusted to 18-bit quantization to match the multiply–accumulation operations in the fully connected layer. Through this transformation, we obtain Equation (9), where the term $M\;=\;\frac{S_{X}S_{W_{1}}}{S_{Y^{1}}}$ is the sole floating-point component. We then use bit-shift operations to approximate this term (*M*) to maintain integer-only computations, as shown in Equation (10). Specifically, we convert the floating-point term into a positive integer $M_{0}$, followed by a right bit-shift operation by *n* positions to approximate the original floating-point value. This integer-only computation is consistently applied to the second hidden layer, as indicated in Equation (11), ensuring uniform integer-only processing throughout our model.

(7) $$S_{Y^{1}}\left(Y_{q}^{1}-Z_{Y^{1}}\right)\approx S_{X}\left(X_{q}-Z_{X}\right)S_{W^{1}}\left(W_{q}^{1}-Z_{W^{1}}\right)+S_{B^{1}}B_{q}^{1}$$

(8) $$S_{Y^{1}}\left(Y_{q}^{1}-Z_{Y^{1}}\right)\approx S_{X}\left(X_{q}-Z_{X}\right)S_{W^{1}}\left(W_{q}^{1}-Z_{W^{1}}\right)+S_{X}S_{W^{1}}B_{q}^{1*}$$

(9) $$Y_{q}^{1}\approx \frac{S_{X}S_{W^{1}}}{S_{Y^{1}}}\left(\left(X_{q}-Z_{X}\right)\left(W_{q}^{1}-Z_{W^{1}}\right)+B_{q}^{1*}\right)+Z_{Y^{1}}$$

(10) $$M=\frac{S_{X}S_{W^{1}}}{S_{Y^{1}}}\approx 2^{-n}M_{0}$$

(11) $$Y_{q}^{2}\approx \frac{S_{Y^{1}}S_{W^{2}}}{S_{Y^{2}}}\left(\left(A_{q}-Z_{Y^{1}}\right)\left(W_{q}^{2}-Z_{W^{2}}\right)+B_{q}^{2*}\right)+Z_{Y^{2}}$$

##### Integer-Only ReLU

Regarding integer-only ReLU, our implementation follows *TensorFlow Lite*’s integer-only ReLU approach, using $Z_{Y^{1}}$ as a threshold, as shown in Equation (12). This threshold aligns with the zero point in the floating-point domain, ensuring that our integer-only ReLU closely replicates the behavior of its floating-point equivalent.

(12) $$A_{q}\approx \max\left(Z_{Y^{1}},Y^{1}\right)$$

### 4.2. Optimized Model Inference on FPGAs

We implemented several optimizations to support integer-only quantized MLP accelerators to run on embedded FPGAs effectively.

#### 4.2.1. Linear Layer Optimization

Our linear layer optimization builds on the foundational designs of [27], adapting VHDL templates to our requirements for integer-only inference. The current VHDL template is specifically optimized to achieve high clock frequencies and efficient resource utilization. This template can be modified to incorporate alternative optimization objectives in the future, such as reduced power consumption or enhanced scalability.

##### Configurable Parameters

The VHDL template in our study incorporates a range of configurable parameters: $M_{0}$, *n*, $Z_{X}$, $Z_{W}$, and $Z_{Y}$. These parameters align closely with our quantization implementation, ensuring a seamless transition from software to hardware. Using the second hidden layer as an example, the parameters $M_{0}$ and *n* are instrumental for bit-shifting operations that hone in on the precise approximation of the scale factor $\frac{S_{Y^{1}}S_{W^{2}}}{S_{Y^{2}}}$. Here, $Z_{X}$, $Z_{W}$, and $Z_{Y}$ align with $Z_{Y^{1}}$, $Z_{W^{2}}$, and $Z_{Y^{2}}$ respectively.

##### Pipelined Matrix Multiplication

Leveraging FPGA parallelization capabilities, we optimized the fully connected layer with pipelined *Arithmetic Logic Unit* (ALU) for matrix multiplication. Key enhancements include (a) zero-point subtraction prior to *Multiplication and Accumulation* (MAC) operations and (b) a pipelined MAC architecture for complex scaling requirements, as shown in Algorithm 1. These changes enable efficient parallel execution, reduced latency, and higher clock frequencies. Steps 7 to 9 separate data fetching and zero-point subtraction from the primary MAC process, while bit-shifting for *M* occurs after computation, optimizing the scaling just before storing results in the output buffer *Y*. Steps 3 to 5 and 12 to 14 are designed to execute within a 100 MHz clock cycle, despite spanning distinct stages.
**Algorithm 1:** MAC Algorithm in the fully connected layer  **Input**: *x* is an *K*-element vector, *W* is an J×K matrix, *B* is an *J*-element vector
  1Initialization: sum←0,j←0;  2**repeat**;  3    k←0;  4    Load: W[j][k],x[k],B[j];  5    sum←sum+B[j];  6    **repeat**;  7       Load: W[j][k+1],x[k+1];  8       $w\leftarrow W\left[j\right]\left[k\right]-Z_{w},x\leftarrow X\left[k\right]-Z_{x}$;  9       sum←sum+w·x;10       k←k+1;11    **until** k=K;12    $y\leftarrow (sum\cdot M_{0})>>n$;13    Store: $Y\left[j\right]\leftarrow y+Z_{y}$;14    j←j+1;15 **until** j=J;       **Output**: *Y*

#### 4.2.2. ReLU Optimization

The ReLU function optimization is designed to handle inputs element-wise, ensuring a straightforward and delay-free operation. Including the configurable parameter $Z_{Y^{1}}$ sets a threshold for the input tensor, enabling the comparator to adjust the output as needed. If the input is less than $Z_{Y^{1}}$, the output is set to this threshold value. Otherwise, it retains the input value. This efficient logic allows for immediate output updates following input changes.

#### 4.2.3. Network Component Integration

Building upon the optimizations of linear layers and ReLU functions, we developed a network component to implement MLP models at the hardware level. This component sequentially interconnects layers with activation functions, replicating the data flow established in the software implementation. However, our prior design [18] was constrained to a fixed four-layer MLP structure, limiting its applicability to more complex or varied deployment scenarios.

To address these limitations, we enhanced the network component (https://github.com/es-ude/elastic-ai.creator/tree/add-linear-quantization/elasticai/creator/nn/integer/sequential accessed on 24 December 2024) to support a wide range of sequential NNs, enabling users to specify any desired layer count. The integration leverages a modular VHDL template, ensuring scalability and efficient resource management as additional layers are added.

**Figure 3:**
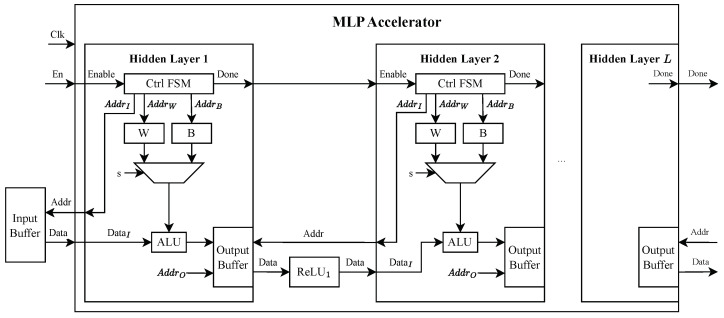
Block Diagram of the generated MLP accelerator.

As depicted in Figure 3, the block diagram illustrates the digital design of the generated MLP accelerator. The design comprises key components such as an input buffer, multiple hidden layers, and activation functions. Each hidden layer is equipped with dedicated memory blocks for weights *W* and biases *B*, control *Finite State Machines* (FSMs) to generate memory addresses, and *Arithmetic Logic Units* (ALUs) to perform matrix multiplications. Following each hidden layer, ReLU activation functions are seamlessly integrated to preserve the sequential flow of data. Output buffers store intermediate results, which are passed to subsequent layers or the final output. This modular design enables the straightforward connection of layers with minimal changes, ensuring scalability while maintaining resource efficiency.

Extensive validation confirmed that our FPGA implementation produces outputs consistent with the software-defined MLP models across various configurations. Although this work focuses on MLP models as a case study, this setup is inherently flexible and can be adapted to other sequential NN architectures.

## 5. End-to-End Workflow and Open-Source Toolchain

**Figure 4:**
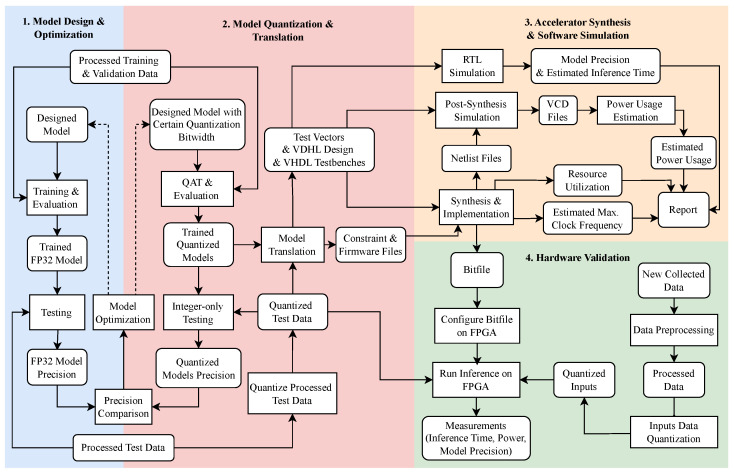
Workflow of end-to-end deployment.

To streamline the development and deployment of efficient MLP-based soft sensors on resource-constrained embedded FPGAs, we propose an end-to-end workflow integrated with our custom open source toolchain, *ElasticAI.Creator*. This toolchain is designed to make the process accessible, enabling even developers without extensive FPGA expertise to implement integer-only quantized models on embedded FPGAs. Figure 4 illustrates the entire workflow, structured into four stages:

1. **Model Design and Optimization in PyTorch**: Users design and train initial FP32 models in PyTorch, utilizing a dataset representative of the target application. This stage focuses on building a robust and accurate baseline model to serve as the foundation for further quantization and deployment, ensuring the model’s adaptability to integer-only processing requirements.
2. **Model Quantization and Translation in ElasticAI.Creator**: Users employ QAT to configure a quantized model mirroring the architecture of the previously trained FP32 model. Depending on specific deployment objectives, the quantized model can be trained from scratch or initialized using the pre-trained FP32 model parameters. After quantization, *ElasticAI.Creator* translates the integer-only quantized model into a set of VHDL files tailored for the corresponding FPGA accelerator.
3. **Accelerator Synthesis and Software Simulation**: The generated VHDL files are subjected to simulation to verify model precision. During the synthesis process, resource usage and power estimation reports are produced, with which we can identify performance bottlenecks and ensure the model aligns with real-time and resource constraints, enabling further fine-tuning to enhance model efficiency.
4. **Hardware Validation**: The bitfile generated during synthesis is deployed onto the selected FPGA. By executing the accelerator on real hardware, inference latency, power usage, and precision are validated to confirm the accelerator’s overall performance.

This workflow simplifies the FPGA deployment process by automating key stages, from quantization to hardware generation, thereby reducing potential errors and manual intervention. The complete workflow, along with example implementations and documentation, is accessible at the *OnDeviceSoftSensorMLP* (https://github.com/Edwina1030/OnDeviceSoftSensorMLP accessed on 24 December 2024) GitHub repository.

## 6. Testbed Platforms and FPGA Comparative Analysis

This chapter presents a detailed overview of the two FPGA-based AI acceleration platforms utilized as experimental testbeds in this study: *Elastic Node V5*, featuring the XC7S15 FPGA, and *Elastic Node V5 SE*, incorporating the iCE40UP5K FPGA. These platforms systematically evaluate the proposed workflow’s effectiveness in generating MLP-based soft sensors tailored to diverse deployment goals.

### 6.1. Elastic Node V5 Hardware Platform

**Figure 5:**
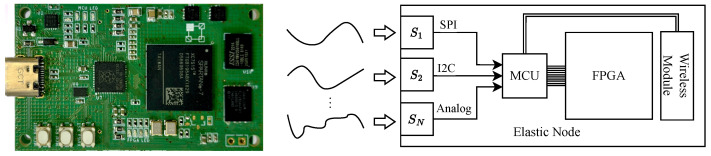
Elastic Node V5 (**left**) and its schematic diagram (**right**).

The *Elastic Node V5*, shown in the left subfigure in Figure 5, provides an on-device AI acceleration environment. It combines an *RP2040 ARM Cortex-M0+* MCU as the controller with an XC7S15 FPGA as the main AI accelerator. As illustrated in the schematic diagram on the right, sensors $S_{1}$ to $S_{N}$ interface with the MCU through digital (e.g., SPI, I2C) or analog channels, and the MCU coordinates data acquisition and manages inference requests to the FPGA. A Microchip *PAC1934* power meter monitors power usage, providing real-time insights for managing energy consumption.

The FPGA remains idle until the MCU initiates it for inference tasks, conserving power until computation is needed. Upon powering-on, the FPGA accelerates model inference, offering a substantial increase in processing speed over the MCU. After model inference, results can be sent to the Cloud, enhancing operational flexibility.

### 6.2. Elastic Node V5 SE Hardware Platform

**Figure 6:**
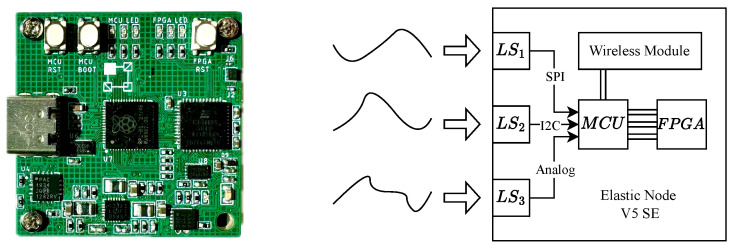
Elastic Node V5 SE (**left**) and its schematic diagram (**right**).

For ultra-low-power applications, we designed the *Elastic Node V5 SE*, a compact variant of the *Elastic Node V5*, as shown in Figure 6. While retaining the *RP2040* MCU, this platform uses the low-power iCE40UP5K FPGA, targeting applications prioritizing extended operating periods over computational capacity. The iCE40UP5K FPGA handles data via an SPI interface to the MCU, with sensor data gathered through I2C or SPI. An MEMS oscillator provides a default 16 MHz clock for low-power operation.

### 6.3. Comparison of FPGA Platforms

Table 2 summarizes the key characteristics of each FPGA, including the specification and amount of Look-Up Tables (LUTs), Block RAMs (BRAMs) on the XC7S15 FPGA, Embedded Block RAMs (EBRs) on the iCE40UP5K, and Digital Signal Processing (DSP). We also attached the cost of each FPGA.

The XC7S15 FPGA, with its extensive resources, provides 12,800 LUTs (6-input), 360 Kbits of BRAMs, and 20 DSP slices operating up to 741 MHz. This architecture allows for highly configurable MLP models with higher layer and neuron counts, supporting applications that demand high precision and throughput. However, its higher power consumption limits its suitability for power-sensitive applications. In contrast, the iCE40UP5K prioritizes energy efficiency with 5280 LUTs (4-input), 120 Kbits of BRAMs, and 8 DSP blocks capped at 50 MHz. Despite its reduced computational resources, it offers a viable option for power cost-sensitive applications, with standby power as low as 86.4 μW compared to the XC7S15’s 36 mW [29,30]. Its lower price (EUR 6.96) makes it suitable for large-scale deployments in low-power environments.

Our prior work [18] showed that a four-layer 8-bit quantized MLP model with 120 neurons per layer on the XC7S15 used only 6.47% of LUTs, 7.5% of BRAMs, and 10% of DSPs. This indicated a possibility for migration to smaller FPGA hardware, like the iCE40UP5K, without sacrificing the deployability. However, a careful re-evaluation of the feasibility of iCE40UP5K’s limited resources is required. Additionally, its DPS’s 50 MHz operating frequency presents challenges for meeting real-time constraints in high-throughput applications.

## 7. Experimental Design

This chapter outlines our experimental setup, covering a case study with corresponding datasets, training settings, and software- and hardware-specific evaluation metrics.

### 7.1. Case Study and Datasets

Fluid flow measurement are critical in numerous industrial and environmental applications [31,32]. Accurate flow rate measurements are essential for optimizing operational efficiency, improving resource allocation, and ensuring safety in areas such as sewer system management [33,34], chemical manufacturing [35], and resource extraction [36]. Despite the widespread use of physical sensor-based techniques, significant challenges persist [37,38]. Contact-based flow meters, though effective, are susceptible to wear and fouling in harsh environments, necessitating frequent maintenance. Non-contact sensors reduce these issues but often lack the precision required for high-stakes management applications [39]. Advanced sensor technologies, such as *Coriolis* and *Magnetic Induction* devices, offer high precision but are prohibitively expensive, limiting their widespread use.

As a cost-effective alternative, soft sensors provide a viable solution for fluid flow measurement by estimating flow rates from auxiliary measurements, such as fluid level data, processed through mathematical models. This approach reduces reliance on costly, maintenance-intensive hardware. Prior research has highlighted the potential of soft sensors for fluid level-based flow estimation [37,39]. For example, Noori et al. [37] employed a *Venturi* structure within a drilling fluid circulation system, where level sensors captured input data, and a *Coriolis* mass flow meter provided target data for calibration. A simple MLP model was then utilized to efficiently estimate non-Newtonian fluid flow, demonstrating the feasibility of MLP-based soft sensors for fluid flow applications.

**Figure 7:**
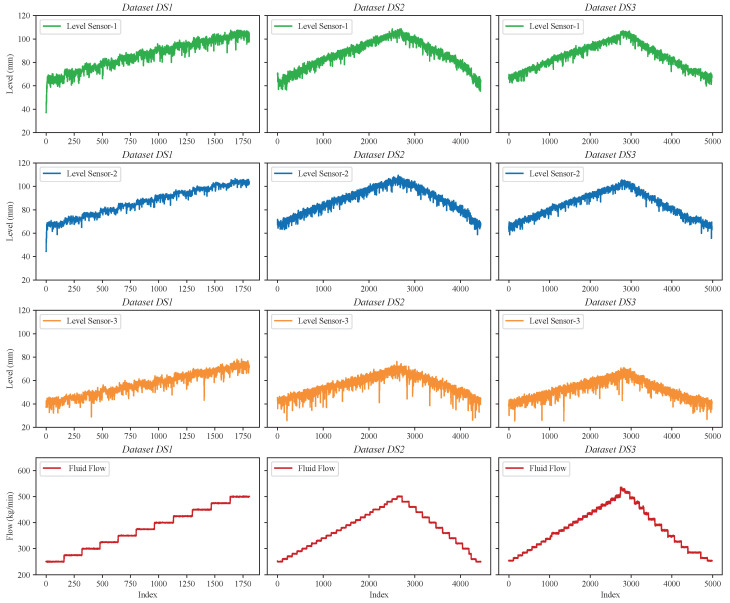
Visualization of three datasets (*DS1*, *DS2*, and *DS3*).

In our study, we build upon this approach, using fluid flow estimation as a case study to explore the deployment of integer-only quantized MLP models on embedded FPGAs. We utilized three distinct datasets, summarized in Table 3. *DS1* is an open dataset referenced from [37]. This system includes a mud tank and a Venturi structure, with high-fidelity data captured at a 10 kHz sampling rate from three level sensors. The data from these sensors serve as input, while a precise Coriolis mass flow meter provides the target data, as depicted in the left subfigure in Figure 7. To further strengthen our model evaluation, we incorporated two additional datasets, *DS2* and *DS3*, generously provided by Viumdal, a co-author of [37]. These datasets are characterized by upward and downward flow trends, introducing additional complexities and enhancing the comprehensiveness of our model’s assessment, as illustrated in the middle and right subfigures in Figure 7.

For all datasets, we maintained the same data partitioning: 75% for training, with the remaining 25% equally split between validation and testing. Additionally, to ensure a robust evaluation of the model’s generalization capabilities, we employed 7-fold cross-validation across all datasets. All data were normalized to a range between 0 and 1 before training.

### 7.2. Training Settings

Each model configuration was trained over 100 sessions, each consisting of up to 500 epochs, with early stopping implemented to mitigate overfitting. The early stopping criterion was based on validation loss, with a patience threshold of 10 epochs. We used a batch size of 100 and the Adam optimizer with standard parameters ($\beta_{1}\;=\;0.9$, $\beta_{2}\;=\;0.98$, $\epsilon \;=\;10^{-9}$) and an initial learning rate of 0.001. Training sessions were conducted on an NVIDIA GeForce RTX 2080 SUPER GPU, utilizing CUDA 11.0 and PyTorch 3.11 on the Ubuntu operating system. The mean squared error was used as the loss function.

### 7.3. Evaluation Metrics

We defined evaluation metrics in two categories: model precision metrics and hardware evaluation metrics.

#### 7.3.1. Model Precision Metrics

To evaluate the predictive precision of the trained models, we used two metrics:

- **Mean Squared Error (MSE)**: Defined in Equation (13), MSE calculates the average squared deviation between predictions (${\hat{y}}_{i}$) and target values ($y_{i}$), offering a scale-sensitive measure of precision.
  (13) $$\mathrm{MSE}=\frac{1}{n}\sum_{i=1}^{n}{\left({\hat{y}}_{i}-y_{i}\right)}^{2}$$
- **Mean Absolute Percentage Error (MAPE)**: Defined in Equation (14), MAPE measures average absolute percentage differences between predictions (${\hat{y}}_{i}$) and targets ($y_{i}$), providing a scale-independent assessment.
  (14) $$\mathrm{MAPE}=\frac{100}{n}\sum_{i=1}^{n}\left|\frac{y_{i}-{\hat{y}}_{i}}{y_{i}}\right|$$

We computed both metrics on denormalized test data to ensure that the predictions and ground truth values are evaluated in their original scale, providing more interpretable performance results.

#### 7.3.2. Hardware Evaluation Metrics

Our hardware evaluation metrics encompass resource usage, inference time, power, and energy consumption across FPGAs.

##### Resource Usage

Resource usage was evaluated by analyzing the consumption of LUTs and DSPs for each generated accelerator across both FPGA platforms. Furthermore, platform-specific memory resources were examined, distinguishing between EBRs on the iCE40UP5K and BRAMs on the XC7S15.

##### Inference Time

Inference time quantifies the duration required for completing a forward pass through the integer-only quantized MLP accelerator deployed on an FPGA. It is computed using Equation (15), where $C_{\mathrm{model}}$ represents the total clock cycles consumed by the accelerator during execution. These clock cycles are determined through VHDL simulations conducted in GHDL, with the final value obtained by averaging the results of five independent inference runs to ensure reliability. Additionally, $f_{\mathrm{clock}}$ denotes the clock frequency of the FPGA. For this study, the clock frequency is configured at 100 MHz for the XC7S15 and 16MHz for the iCE40UP5K.

(15) $$T_{\mathrm{inference}}=\frac{C_{\mathrm{model}}}{f_{\mathrm{clock}}}$$

##### Power and Energy Consumption

Power consumption was evaluated by measuring both static and dynamic components on each FPGA platform. Static power represents the baseline consumption of the FPGA in an idle state, while dynamic power accounts for the additional energy required during active model inference. Energy consumption per inference was calculated by multiplying the total power values with the corresponding inference time, as described in Equation (16). In this equation, $E_{\mathrm{inference}}$ denotes the energy consumed per inference, and $P_{\mathrm{total}}$ represents the combined static and dynamic power consumption.

(16) $$E_{\mathrm{inference}}=P_{\mathrm{total}}\times T_{\mathrm{inference}}$$

## 8. Results and Analysis

This chapter provides a detailed evaluation of the selected case study, focusing on model precision and hardware efficiency metrics across two FPGA platforms: the XC7S15 and iCE40UP5K. Initially, we analyze the precision of FP32 models by varying the layer and neuron count across three datasets, establishing baseline precision metrics for subsequent comparisons. Next, we assess the effect of different quantization bitwidths on model precision under a consistent configuration. Finally, we evaluate the performance of the generated accelerators by comparing resource usage, inference time, power consumption, and energy consumption on both FPGAs.

### 8.1. Experiments 1: FP32 Model Analysis

**Figure 8:**
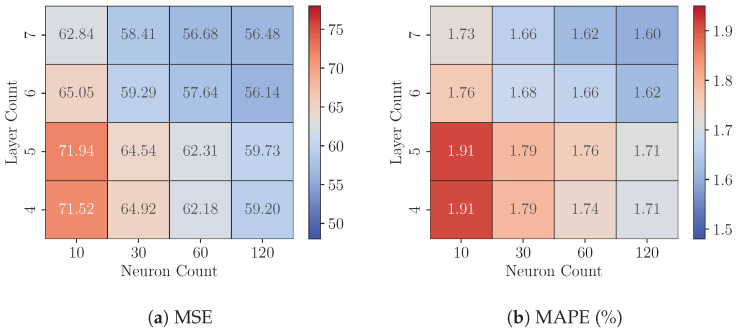
On Dataset *DS1*: Performance of FP32 models with varying configurations.

**Figure 9:**
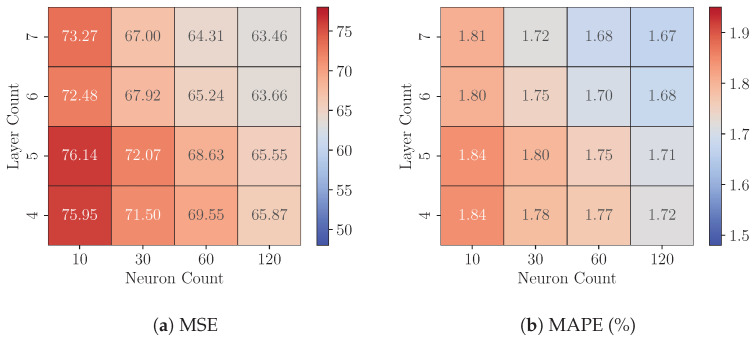
On Dataset *DS2*: Performance of FP32 models with varying configurations.

**Figure 10:**
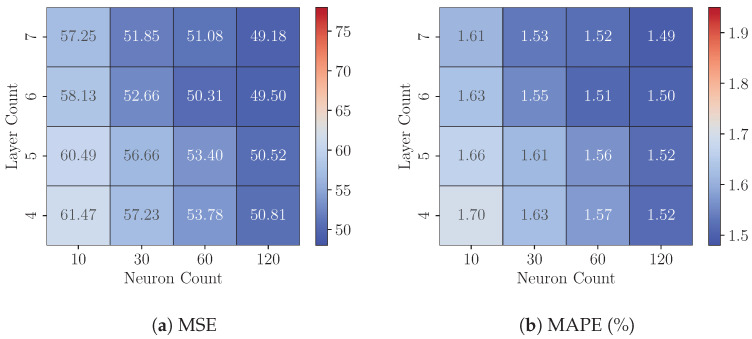
On Dataset *DS3*: Performance of FP32 models with varying configurations.

This experiment examines the impact of model complexity on predictive precision by analyzing variations in the layer count (ranging from 4 to 8) and the neuron count per hidden layer (ranging from 10 to 120) in FP32 MLP models across three datasets (*DS1*, *DS2*, and *DS3*). Figure 8, Figure 9 and Figure 10 present each dataset’s Test MSE and MAPE, respectively. These heatmaps represent the average best-achieved metrics across seven folds for various configurations.

For a fixed layer count, increasing the neuron count consistently reduces Test MSE and MAPE across all three datasets, demonstrating that models with more neurons capture complex data relationships more effectively. The precision gains generally range from a 5.35% to 17.34% reduction in MSE. For instance, on dataset *DS3*, increasing the neuron count from 10 to 120 in a four-layer model lowers the Test MSE from 61.47 to 50.81, a 17.34% improvement.

However, the effect of adding more layers is mixed. Increasing the layer count significantly improves precision for configurations with fewer neurons (e.g., 10 or 30), as evidenced by notable reductions in MSE and MAPE. This trend is particularly evident on dataset *DS1*. For instance, with 10 neurons, increasing the layer count from four to seven reduces the MSE from 71.52 to 62.84, a 12.13% improvement. Conversely, as the neuron count increases, the marginal benefits of adding more layers diminish, indicating a saturation of the model’s representational capacity. For instance, on dataset *DS3*, when the neuron count is 120, increasing the layer count from four to seven results in a smaller reduction in MSE, from 50.81 to 49.18, representing a modest 3.21% improvement.

Among the three datasets, dataset *DS3* consistently achieves the lowest MSE and MAPE values, suggesting that it has a more predictable structure or lower noise levels, making it easier for the models to generalize. In contrast, dataset *DS2* yields the highest MSE values across all configurations, indicating that its underlying patterns may be more complex or noisy, posing greater challenges for accurate prediction. These results underscore the importance of dataset-specific tuning of model complexity to achieve optimal performance.

While increasing layer count and neuron count generally enhances precision, these gains come at the expense of computational and memory demands. Later experiments will quantify these resource requirements on FPGA platforms, providing insights into the balance between model precision and hardware efficiency for resource-limited deployments.

### 8.2. Experiments 2: Quantized Models Analysis

In this experiment, we assess the effect of quantization on model precision, specifically focusing on 8-bit, 6-bit, and 4-bit quantization across the model configurations explored in Experiment 1. The analysis is conducted across three datasets (*DS1*, *DS2*, and *DS3*), with *DS1* selected as the representative example due to similar trends observed across all datasets.

**Figure 11:**
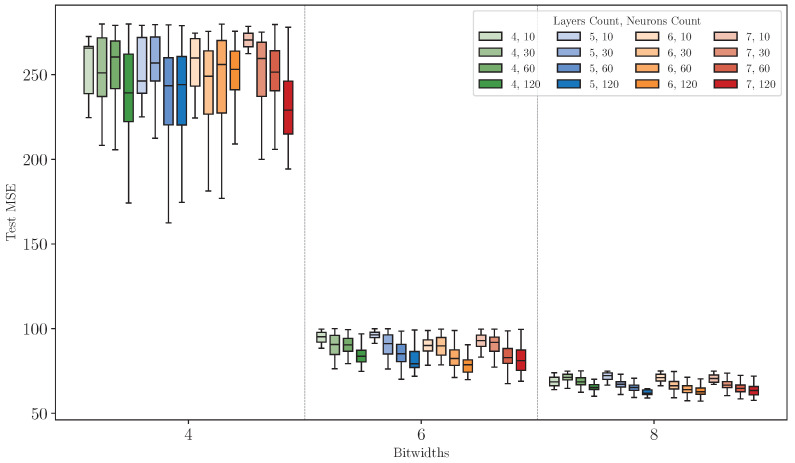
MSE distribution of quantized models with varying configurations on dataset *DS1*.

Figure 11 illustrates the Test MSE distribution of quantized models with various configurations across different quantization bitwidths. The results highlight that bitwidth is the dominant factor influencing the performance of quantized models. Models quantized at 4-bit exhibit significantly higher Test MSE values and a broader distribution than those quantized at 6-bit or 8-bit, indicating a substantial loss introduced by lower bitwidths. As bitwidth increases, the Test MSE distribution narrows, particularly for 8-bit models, which achieve performance levels close to the FP32 benchmark.

The neuron count significantly impacts the Test MSE under quantization. Models with a larger neuron count (e.g., 120 neurons) generally demonstrate better performance, with lower median Test MSE values and more centralized distributions. This improvement is particularly evident under 6-bit and 8-bit quantization, showing that larger models are better equipped to absorb quantization noise. However, under 4-bit quantization, the relationship becomes less predictable. While configurations with higher neuron count occasionally achieve better median precision, their overall distribution widens, indicating less stability and a reduced ability to absorb errors introduced by low bitwidth quantization consistently.

The layer count has a more mixed effect on performance. In deeper models (e.g., six or seven layers), the Test MSE distribution under 4-bit quantization becomes narrower, with lower medians than shallower models (e.g., four or five layers). This suggests that deeper models are better equipped to handle the errors introduced by quantization, leveraging their additional complexity to absorb and mitigate quantization noise. In contrast, shallower models exhibit broader and less consistent Test MSE distributions under lower bitwidths, underscoring their vulnerability to quantization-induced errors. Interestingly, this contrasts with the findings from Experiment 1, where increasing the number of layers showed diminishing returns in precision. Here, deeper models demonstrate a distinct advantage in maintaining performance robustness, particularly under 4-bit quantization.

**Figure 12:**
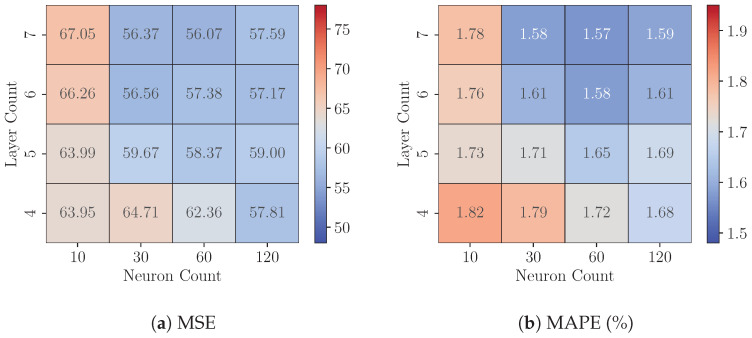
Performance of best-precise 8-bit quantized models for each configuration.

**Figure 13:**
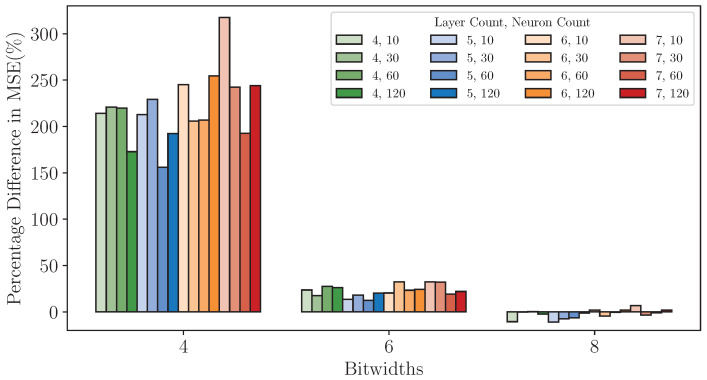
Percentage difference in MSE across various model configurations and bitwidths on *DS1*.

We then identified the best-precise model under each configuration for subsequent experiments on the FPGA platform. Figure 12 shows that 8-bit quantized models exhibit exceptional performance. For instance, the seven-layer, 120-neuron configuration achieves a Test MSE of 57.59 and a Test MAPE of 1.59%, demonstrating its suitability for precision-critical applications. Even smaller configurations, such as the 4-layer, 10-neuron model, maintain strong performance with a Test MSE of 63.95 and a Test MAPE of 1.82%, further highlighting the robustness of 8-bit quantization across diverse model sizes. Compared to their FP32 counterparts, 8-bit models show mixed results of percentage difference in MSE, as evidenced by Figure 13. Among the 16 configurations, five exhibit higher MSE values under 8-bit quantization, with deviations ranging from 0.28% to 6.69%, while the remaining 11 configurations achieve reduced MSE values, with improvements ranging from 0.32% to 11.04%.

Notably, no consistent pattern emerges regarding the sensitivity of model configurations to 8-bit quantization. While smaller models might be expected to exhibit greater vulnerability to quantization noise due to their lower representational capacity, this trend is not universally observed. For example, the four-layer, 10-neuron model achieves better Test MSE performance under 8-bit quantization (−10.58%), while the six-layer, 120-neuron model shows a slight increase (+1.84%). This lack of a clear correlation suggests that other factors, such as the interplay between layer count, neuron count, and the inherent noise resilience of the dataset, may play a significant role in determining the quantization impact. In general, these findings reaffirm the effectiveness of 8-bit quantization for most configurations, with precision losses being minimal and occasionally yielding improvements due to potential regularization effects introduced by quantization noise.

**Figure 14:**
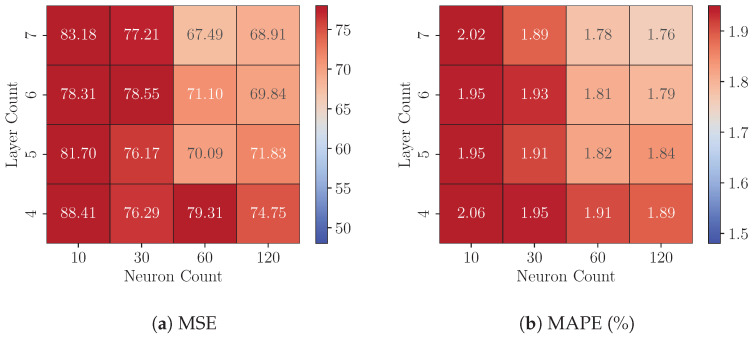
Performance of best-precise 6-bit quantized models for each configuration.

At 6-bit quantization, models generally maintain acceptable performance, but the reduction in bitwidth introduces a more pronounced degradation in precision compared to 8-bit quantization. As shown in Figure 14, the Test MSE and Test MAPE values increase across nearly all configurations relative to their 8-bit counterparts. For instance, the seven-layer, 120-neuron model now has a Test MSE of 68.91 and a Test MAPE of 1.76%, representing an increase of 19.65% in Test MSE compared to the 8-bit version. Smaller models are also obviously affected. The four-layer, 10-neuron model exhibits a Test MSE of 88.41 and a Test MAPE of 2.06%, a substantial increase from its performance at 8-bit quantization. Figure 13 further highlights the differences in Test MSE between the 6-bit models and their FP32 counterparts. Across all configurations, the percentage difference in Test MSE ranges from 12.48% to 32.36%. However, as with the 8-bit results, the effects of model complexity (i.e., layer count and neuron count) on quantization sensitivity remain inconsistent. Despite these challenges, Test MAPE values remain around 2% for all configurations, indicating that 6-bit quantization can still meet industrially acceptable precision requirements. This makes it a practical choice for scenarios where resource efficiency is a priority, providing a viable trade-off between precision and computational cost.

**Figure 15:**
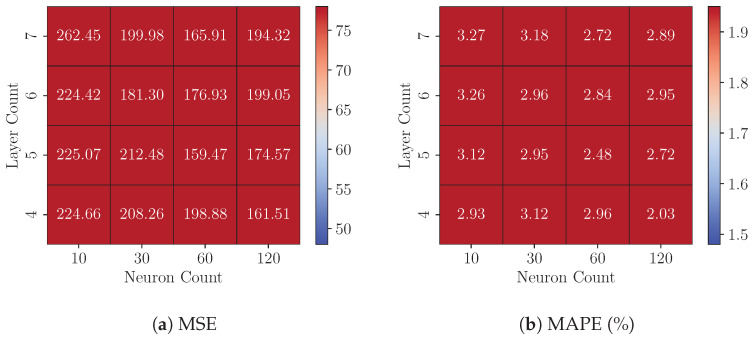
Performance of best-precise 4-bit quantized models for each configuration.

At 4-bit quantization, the effects of reduced precision become far more pronounced, leading to significant increases in Test MSE and MAPE across configurations. Figure 15 reveals that most configurations exhibit considerable performance degradation. For instance, the seven-layer, 120-neuron model now has a Test MSE of 194.32 and a Test MAPE of 2.89%, a stark contrast to its 8-bit counterpart. Similarly, smaller models, such as the four-layer, 10-neuron configuration, show a Test MSE of 224.66 and a Test MAPE of 3.27%, indicating that the lower bitwidth severely impacts precision. The percentage differences in Test MSE compared to FP32 models (Figure 13) further underscore the significant impact of 4-bit quantization. Although Test MAPE values remain below 4% in all configurations, the overall increase in Test MSE indicates that 4-bit quantization is only suitable for applications where resource constraints outweigh the need for precision.

### 8.3. Experiments 3: Cross-Platform Performance Comparison

In this third set of experiments, we deploy the best-precise models identified in Section 8.2, which achieve the lowest test MSE for each configuration, onto two distinct FPGA platforms: the XC7S15 and iCE40UP5K, using dataset *DS1* as an example. The objective is to comprehensively evaluate these configurations across multiple dimensions, including resource usage, inference time, power consumption, and energy efficiency, while considering quantization bitwidth, layer count, and neuron count variations. Analyses were conducted using AMD Vivado for the XC7S15 and Lattice Radiant for the iCE40UP5K. To ensure the practicality of our findings, all deployable accelerators were tested on actual hardware, validating the simulation results and providing insights into real-world performance. Based on these results, we provided a comprehensive deployment analysis, offering tailored recommendations for different application scenarios.

#### 8.3.1. Resource Usage Analysis

**Figure 16:**
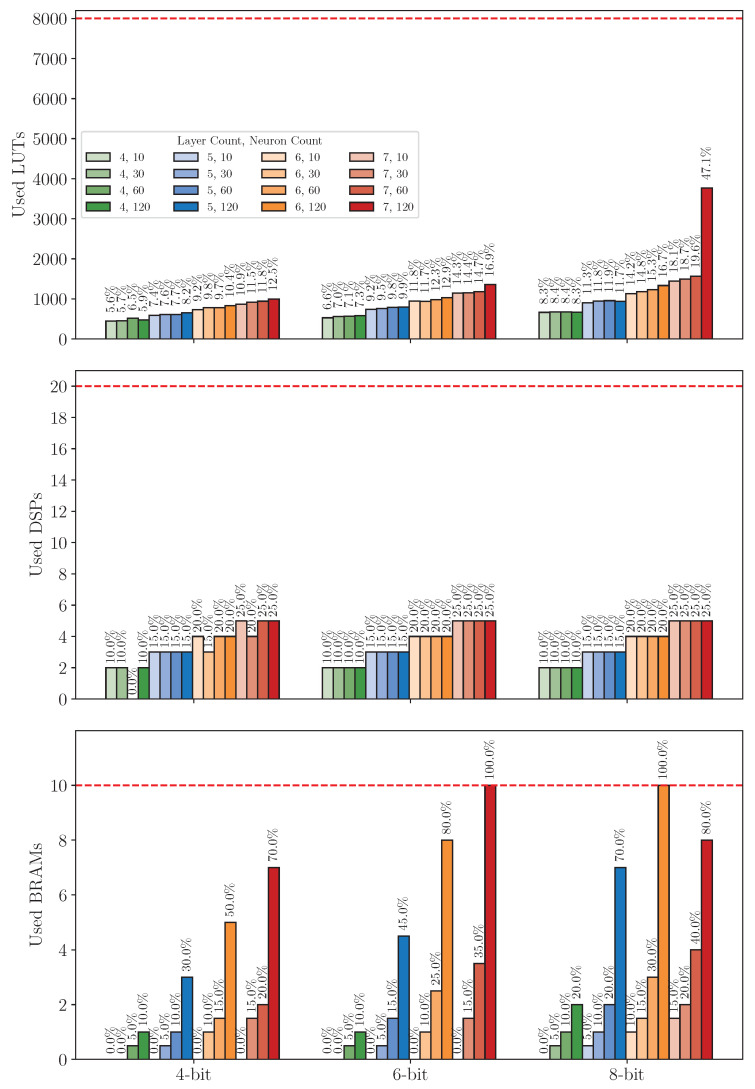
Resource usage on XC7S15 across model configurations on dataset *DS1*.

**Figure 17:**
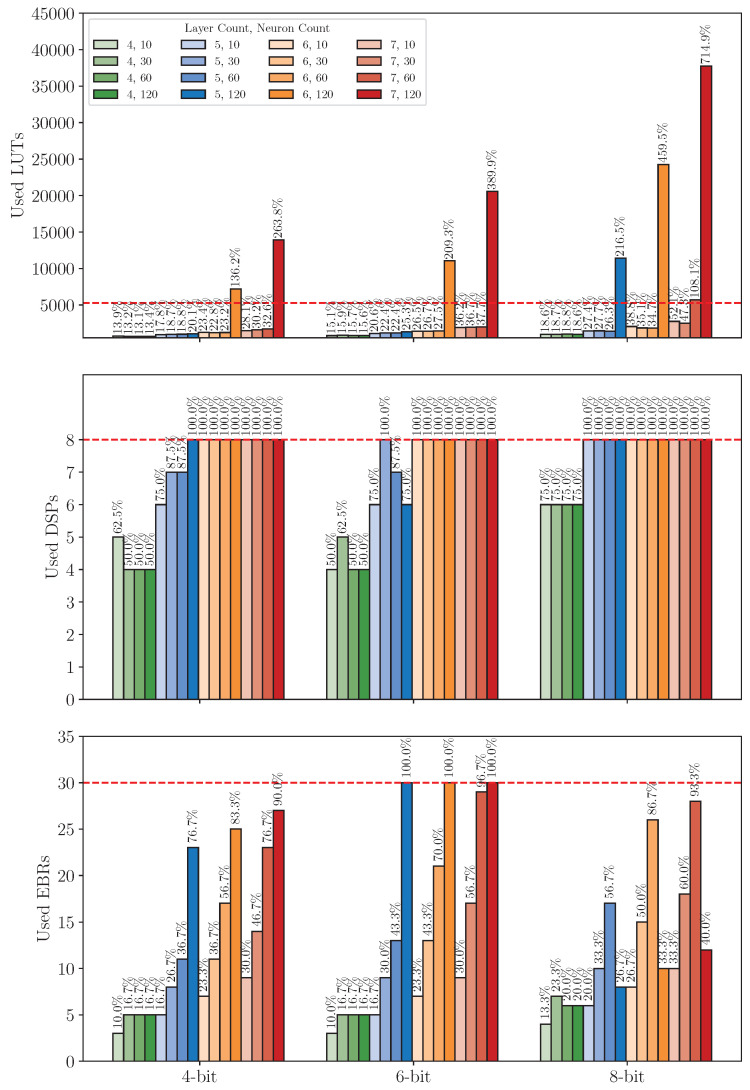
Resource usage on iCE40UP5K across model configurations on dataset *DS1*.

Figure 16 and Figure 17 illustrate the resource usage for LUTs, DSPs, and BRAMs/EBRs across various configurations on the XC7S15 and iCE40UP5K FPGAs, respectively. The bar heights represent the absolute resource usage, while the corresponding percentages displayed on top of each bar indicate the resource utilization relative to the platform’s capacity. Additionally, the horizontal red dashed lines mark the maximum resource limit, providing a clear visual reference for checking whether a configuration is exceeding available resources.

On the XC7S15 (see Figure 16), LUTs utilization generally increases with higher neuron count, layer count, and larger bitwidth, and the exact increases depend on specific configurations. The DSPs utilization exhibits a consistent trend, increasing proportionally with layer count while remaining largely unaffected by neuron count or bitwidth. This behavior aligns with the design specification, as mentioned in Section 4.2.1, where each layer instantiates a single ALU to loop through the MAC operations. As a result, the DSPs resource consumption per layer is independent of neuron count or bitwidth. The BRAMs utilization also shows an expected increase with higher layer count, larger neuron count, and bigger bitwidth.

However, some configurations deviate from these trends, particularly at 4-bit quantization. For instance, in the 4-layer accelerator with 60 neurons at 4-bit quantization, the DSPs utilization unexpectedly drops to 0. In contrast, its LUTs utilization increases to 6.5%, surpassing the LUTs utilization of a comparable four-layer accelerator with 120 neurons, which exhibits only 5.9% LUTs utilization. A similar anomaly is observed in the six-layer accelerator with 30 neurons under 4-bit quantization. These deviations are consistent with the design flexibility of the hardware, where ALUs can be implemented using either DSPs or LUTs. At 4-bit quantization, implementing ALUs with LUTs does not significantly affect the accelerator’s timing performance, providing a viable alternative to DSPs.

In addition, a notable pattern is also observed in the seven-layer accelerator with 30 neurons at 4-bit quantization, where the DSPs utilization decreases to 20% compared to the 25% utilization observed in the seven-layer accelerator with higher neuron count. In this case, the LUTs utilization does not increase as expected. However, the BRAMs utilization rises significantly to 15%, the same level observed for the seven-layer accelerator with 30 neurons at 6-bit quantization. This behavior aligns with Vivado’s automatic storage selection mechanism, where intermediate results and model parameters are allocated to either BRAMs or LUTs, depending on availability.

Moreover, another noteworthy pattern is the underutilization of BRAMs and the overutilization of LUTs in the 8-bit quantized seven-layer accelerator with 120 neurons. Despite the BRAMs utilization dropping to 80%, the LUTs utilization increases significantly. This phenomenon can be attributed to BRAMs’ fixed allocation in chunks and the inability to combine BRAMs and LUTs to construct a single memory instance. When a configuration exceeds the available BRAMs capacity, Vivado compensates by mapping storage requirements to LUTs, resulting in higher LUTs utilization.

On the iCE40UP5K, the general resource usage trend mirrors that of the XC7S15. However, since each EBRs and LUTs block on the iCE40UP5K is relatively smaller compared to those on the XC7S15, the number of blocks utilized is nearly doubled, resulting in approximately 2× higher LUTs and EBRs utilization on the iCE40UP5K. Similarly, the DSPs on the iCE40UP5K, which support only 16-bit input multiplication and 32-bit accumulation, result in around 2× higher DSPs utilization than on the XC7S15, where the DSPs can handle 25-bit by 18-bit input multiplication with up to 48-bit accumulation.

Furthermore, some configurations exceed the iCE40UP5K’s on-chip resource limits. Specifically, even with 4-bit quantization, configurations with six or seven layers and 120 neurons cannot fit within the available resources. At 8-bit quantization, additional configurations, such as the five-layer accelerator with 120 neurons and the seven-layer accelerator with 60 neurons, also exceed the platform’s resource constraints due to excessive LUT requirements. In total, 7 out of 48 configurations exceeded the on-chip resource limit of the iCE40UP5K.

#### 8.3.2. Timing Analysis

Following the findings from Section 8.3.1, we performed timing analysis on the deployable accelerators for both FPGAs. For the XC7S15, the clock frequency was constrained to 100 MHz, while the iCE40UP5K operated at 16 MHz. As shown in Table 4, the substantial difference in operating frequencies naturally results in a proportional performance gap between the two platforms. For instance, a seven-layer accelerator with 120 neurons quantized to 8 bits achieves an inference time of 122.60 μs on the XC7S15, compared to 766.25 μs on the iCE40UP5K, around 6.25× slower, which closely reflects the scaling dictated by the clock speed disparity.

The inference time is linearly proportional to the layer and neuron count, a trend consistent with the architecture design. In this implementation, a single ALU is instantiated per layer, processing operations sequentially across time steps, with layers also executed sequentially. This design results in a clear dependency of inference latency on model complexity, highlighting the trade-offs between layer count, neuron count, and real-time performance when deploying accelerators on resource-constrained platforms like the iCE40UP5K. To ensure the accuracy of our simulation-based timing analysis, we validated the inference time on actual hardware. The measured results demonstrated less than 0.5% deviation from the timing reports generated by AMD Vivado and Lattice Radiant, confirming the reliability of the simulated results for predicting real-world performance.

#### 8.3.3. Power and Energy Analysis

As displayed in Table 4, when analyzing power consumption, accelerators implemented on the iCE40UP5K consistently demonstrate less total power consumption than those on the XC7S15, owing to the platform’s ultra-low-power design. Specifically, the static power consumption of accelerators on the iCE40UP5K ranges from 0.73 to 1.15 mW across all deployable configurations, significantly lower than that on XC7S15, where their static power draw of 30 to 31 mW. In addition, dynamic power consumption further highlights differences between the two FPGA platforms. On the XC7S15, dynamic power scales substantially with increasing model complexity, including higher layer count, larger neuron count, and greater quantization bitwidth, whereas on the iCE40UP5K, dynamic power remains relatively stable across configurations. For example, on the XC7S15, an accelerator implementing a four-layer, 10-neuron model quantized to 4 bits consumes 5 mW in dynamic power. By contrast, a more complex accelerator implementing a seven-layer, 60-neuron model quantized to 6 bits consumes 15 mW—a 200% increase. Meanwhile, the same accelerators deployed on the iCE40UP5K show only a modest rise in dynamic power, from 1.16 mW to 1.30 mW, representing just a 10.7% increase. These findings underscore the iCE40UP5K’s suitability for low-power applications.

**Figure 18:**
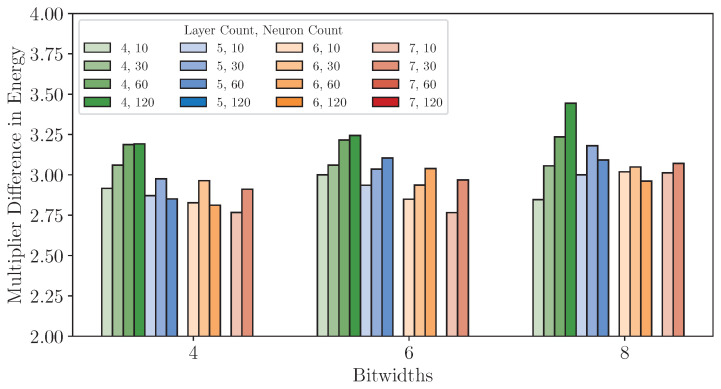
Multiplier difference (the ratio of the energy usage of accelerators on the XC7S15 to those on the iCE40UP5K) in energy consumption across varying deployable configurations on Dataset *DS1*.

The energy consumption per inference provides deeper insights into the trade-offs between the two platforms, as shown in Table 4. For instance, a four-layer, 10-neuron accelerator at 4-bit quantization achieves the lowest energy consumption on the iCE40UP5K, consuming only 0.012 μJ per inference, compared to 0.035 μJ on the XC7S15. Figure 18 further compares the energy consumption of accelerators deployed on two platforms, calculated as the ratio of the energy usage of accelerators on the XC7S15 to those on the iCE40UP5K. The results reveal that the accelerators on the iCE40UP5K consistently achieve significant energy savings, with reductions ranging from 2.77× to 3.44×. This advantage persists across all configurations, reaffirming the iCE40UP5K’s suitability for energy-critical applications. Even for larger accelerators with higher layer count and neuron count, where energy consumption typically increases, the iCE40UP5K maintains its substantial efficiency advantage.

#### 8.3.4. Deployment Analysis

The above experimental results provide verified insights for tailoring FPGA deployment strategies based on specific application requirements. The XC7S15 is highly suitable for high-precision and low-latency scenarios due to its powerful DSP slices, which significantly accelerate inference, even for complex models. However, its higher power consumption necessitates careful consideration in energy-constrained systems. In contrast, the iCE40UP5K is optimized for ultra-low-power applications, such as wearable or battery-operated devices. Its energy-efficient design makes it ideal for accelerators of moderate complexity, although it faces limitations when handling high-complexity configurations. This trade-off underscores the suitability of the iCE40UP5K for energy-critical deployments.

For the specific use case in our study, where real-time constraints demand an inference time below 100 μs to match the 10 kHz sensor sampling frequency, both platforms offer viable options. On the XC7S15, all four-layer accelerators, most five-layer and six-layer accelerators, and half of the seven-layer accelerators meet the timing requirement. Considering precision from Experiment 2, the six-layer, 60-neuron accelerator quantized to 8 bits emerges as an optimal choice, achieving a Test MSE of 56.56, a Test MAPE of 1.61%, and a latency of 23.87 μs, with an energy consumption of 1.003 μJ per inference. This configuration is particularly well-suited for latency-sensitive scenarios requiring high precision.

On the iCE40UP5K, all four-layer accelerators, five-layer accelerators with up to 30 neurons, and six-layer and seven-layer accelerators with 10 neurons satisfy the timing constraint. Among these, the five-layer, 30-neuron accelerator quantized to 8 bits offers a compelling balance between performance and energy efficiency, achieving an inference latency of 83.37 μs and consuming only 0.172 μJ per inference. While this configuration exhibits slightly lower precision (Test MSE of 59.67 and Test MAPE of 1.71%) compared to more complex models, it remains acceptable for applications prioritizing low power consumption and extended operational lifetime.

## 9. Conclusions and Future Work

This paper presents a comprehensive workflow for developing and deploying MLP-based soft sensors on embedded FPGAs, targeting diverse deployment goals, including high precision, low inference latency, reduced power consumption, and improved energy efficiency. We investigated the impact of model configuration factors—including layer count, neuron count, and quantization bitwidth—on the deployability of integer-only quantized MLP models across two distinct FPGA platforms: the XC7S15 and the iCE40UP5K. Our experiments highlighted that, while the XC7S15 can support more complex models with larger layer and neuron counts, it comes at a higher power cost, making it more suitable for applications where precision and timing performance take precedence. In contrast, the iCE40UP5K, though limited in hardware resources, demonstrated significant power and energy savings, especially under lower bitwidth quantization (4-bit and 6-bit), making it ideal for low-power applications where energy efficiency is paramount. This distinction in deployment suitability underscores the importance of matching platform capabilities with application requirements.

For future work, we plan to explore mixed-precision quantization, where different layers or operations within the model are quantized at different bitwidths. This approach could further optimize resource usage and energy consumption, maintaining acceptable levels of precision across layers with different representational needs. Additionally, while this study focused on MLP architectures, extending similar quantization and deployment strategies to other architectures, such as RNNs or *Transformers*, could provide broader insights. These architectures, often required for more complex AI tasks, stand to benefit from FPGA-optimized quantization techniques tailored for real-time, embedded applications in constrained environments.

## Figures and Tables

**Table 1:** Quantization description of a four-layer MLP model.

Layers	Quantization Objects	Quantization Parameters
Input Layer	*X*	$S_{X}$, $Z_{X}$
Hidden Layer 1	$W^{1}$	$S_{W^{1}}$, $Z_{W^{1}}$
	$B^{1}$	$S_{B^{1}}$
	$Y^{1}$	$S_{Y^{1}}$, $Z_{Y^{1}}$
	*A*	$S_{Y^{1}}$, $Z_{Y^{1}}$
Hidden Layer 2	$W^{2}$	$S_{W^{2}}$, $Z_{W^{2}}$
	$B^{2}$	$S_{B^{2}}$
	$Y^{2}$	$S_{Y^{2}}$, $Z_{Y^{2}}$
Output Layer	$Y^{2}$	$S_{Y^{2}}$, $Z_{Y^{2}}$

**Table 2:** Comparison of XC7S15 and iCE40UP5K FPGAs.

		XC7S15 [28]	iCE40UP5K [29]
LUTs	Type	LUT6	LUT4
	Count	12,800	5280
	Total size (Kbits)	360	120
BRAMs/EBRs	Blocks	10	30
DSPs	Width (bits)	25 × 18 + 48	16 × 16 + 32
	Maximum frequency (MHz)	741	50
	Count	20	8
Price (€)		22.58	6.96

**Table 3:** Flow estimation datasets.

Datasets	Description
*DS1*	1800 samples with upward trend only
*DS2*	4439 samples with upward and downward trends
*DS3*	4985 samples with upward and downward trends

**Table 4:** Performance of deployable accelerators across configurations and FPGAs on dataset *DS1*.

FPGAs	Layer Count	Neuron Count	Bitwidth	Clock Cycles	Time (μs)	Power (mW)			Energy (μJ)
						**Static**	**Dynamic**	**Total**	
XC7S15 @100 MHz	4	10	4	10,100	1.01	30.0	5.0	35.0	0.035
			6	10,100	1.01	30.0	5.0	35.0	0.035
			8	10,100	1.01	30.0	6.0	37.0	0.037
		30	4	28,100	2.81	30.0	5.0	36.0	0.101
			6	28,100	2.81	30.0	6.0	36.0	0.101
			8	28,100	2.81	30.0	9.0	39.0	0.110
		60	4	55,100	5.51	30.0	7.0	37.0	0.204
			6	55,100	5.51	30.0	7.0	38.0	0.209
			8	55,100	5.51	30.0	9.0	40.0	0.220
		120	4	109,100	10.91	30.0	8.0	38.0	0.415
			6	109,100	10.91	31.0	8.0	39.0	0.425
			8	109,100	10.91	31.0	12.0	42.0	0.458
	5	10	4	25,400	2.54	30.0	5.0	35.0	0.089
			6	25,400	2.54	30.0	6.0	36.0	0.091
			8	25,400	2.54	30.0	8.0	39.0	0.099
		30	4	13,340	13.34	30.0	6.0	37.0	0.494
			6	13,340	13.34	30.0	8.0	38.0	0.507
			8	13,340	13.34	30.0	10.0	40.0	0.547
		60	4	44,540	44.54	30.0	7.0	37.0	1.648
			6	44,540	44.54	30.0	9.0	40.0	1.782
			8	44,540	44.54	30.0	12.0	42.0	1.871
		120	4	1,609,400	160.94	30.0	11.0	41.0	6.599
			6	1,609,400	160.94	31.0	15.0	46.0	7.403
	6	10	4	40,700	4.07	30.0	6.0	36.0	0.147
			6	40,700	4.07	30.0	6.0	36.0	0.147
			8	40,700	4.07	30.0	10.0	40.0	0.163
		30	4	238,700	23.87	30.0	8.0	38.0	0.907
			6	238,700	23.87	30.0	9.0	39.0	0.931
			8	238,700	23.87	30.0	12.0	42.0	1.003
		60	4	835,700	83.57	30.0	9.0	39.0	3.259
			6	835,700	83.57	30.0	12.0	42.0	3.510
			8	835,700	83.57	30.0	15.0	45.0	3.761
	7	10	4	56,900	5.60	30.0	6.0	36.0	0.202
			6	56,900	5.60	30.0	8.0	38.0	0.213
			8	56,900	5.60	30.0	12.0	42.0	0.235
		30	4	344,000	34.40	30.0	9.0	39.0	1.342
			6	344,000	34.40	30.0	11.0	41.0	1.410
			8	344,000	34.40	30.0	14.0	44.0	1.514
		60	4	1,226,000	122.60	31.0	10.0	41.0	5.207
			6	1,226,000	122.60	31.0	15.0	45.0	5.517
iCE40UP5K @16 MHz	4	10	4	10,100	6.31	0.73	1.16	1.89	0.012
			6	10,100	6.31	0.73	1.20	1.93	0.012
			8	10,100	6.31	0.76	1.27	2.04	0.013
		30	4	28,100	17.56	0.75	1.13	1.89	0.033
			6	28,100	17.56	0.76	1.13	1.90	0.033
			8	28,100	17.56	0.81	1.23	2.04	0.036
		60	4	55,100	34.44	0.75	1.11	1.86	0.064
			6	55,100	34.44	0.76	1.14	1.90	0.065
			8	55,100	34.44	0.79	1.18	1.97	0.068
		120	4	109,100	68.19	0.75	1.16	1.91	0.130
			6	109,100	68.19	0.76	1.16	1.92	0.131
			8	109,100	68.19	0.78	1.16	1.94	0.133
	5	10	4	25,400	15.88	0.77	1.20	1.97	0.031
			6	25,400	15.88	0.78	1.19	1.97	0.031
			8	25,400	15.88	0.81	1.29	2.10	0.033
		30	4	13,340	83.37	0.81	1.18	1.99	0.166
			6	13,340	83.37	0.84	1.16	2.00	0.167
			8	13,340	83.37	0.87	1.19	2.06	0.172
		60	4	44,540	278.38	0.85	1.22	2.08	0.578
			6	44,540	278.38	0.89	1.17	2.06	0.574
			8	44,540	278.38	0.96	1.21	2.17	0.605
		120	4	1,609,400	1005.88	1.02	1.17	2.19	2.200
			6	1,609,400	1005.88	1.13	1.28	2.40	2.417
	6	10	4	40,700	25.44	0.81	1.24	2.05	0.052
			6	40,700	25.44	0.82	1.27	2.09	0.053
			8	40,700	25.44	0.87	1.25	2.12	0.054
		30	4	238,700	149.19	0.86	1.18	2.05	0.306
			6	238,700	149.19	0.91	1.22	2.13	0.317
			8	238,700	149.19	0.96	1.24	2.20	0.329
		60	4	835,700	522.31	0.95	1.27	2.22	1.159
			6	835,700	522.31	1.02	1.20	2.21	1.155
			8	835,700	522.31	1.11	1.32	2.43	1.270
	7	10	4	56,900	35.00	0.86	1.24	2.10	0.073
			6	56,900	35.00	0.88	1.32	2.19	0.077
			8	56,900	35.00	0.93	1.29	2.22	0.078
		30	4	344,000	215.00	0.93	1.21	2.14	0.461
			6	344,000	215.00	0.99	1.22	2.21	0.475
			8	344,000	215.00	1.04	1.25	2.29	0.493
		60	4	1,226,000	766.25	1.06	1.20	2.25	1.740
			6	1,226,000	766.25	1.15	1.30	2.45	1.880

## Data Availability

No new data were created or analyzed in this study.
